# Association between Frequency Domain Heart Rate Variability and Unplanned Readmission to Hospital in Geriatric Patients

**DOI:** 10.1186/1471-2458-11-137

**Published:** 2011-02-27

**Authors:** Jui-Kun Chiang, Chin-Hua Fu, Terry BJ Kuo, Malcolm Koo

**Affiliations:** 1 Department of Family Medicine, Buddhist Dalin Tzu Chi General Hospital, Chiayi, Taiwan; 2 Department of Biotechnology, Chia Nan University of Pharmacy and Science, Tainan, Taiwan; 3 Department of Neurology, Buddhist Taichung Tzu Chi General Hospital, Taichung, Taiwan; 4 Medical school, Tzu Chi University, Hualien, Taiwan; 5 Institute of Brain Science, National Yang Ming University, Taipei, Taiwan; 6 Dalla Lana School of Public Health, University of Toronto, Canada

## Abstract

**Background:**

An accurate prediction of unplanned readmission (UR) after discharge from hospital can facilitate physician's decision making processes for providing better quality of care in geriatric patients. The objective of this study was to explore the association of cardiac autonomic functions as measured by frequency domain heart rate variability (HRV) and 14-day UR in geriatric patients.

**Methods:**

Patients admitted to the geriatric ward of a regional hospital in Chiayi county in Taiwan were followed prospectively from July 2006 to June 2007. Those with invasive tubes and those who were heavy smokers, heavy alcohol drinkers, on medications that might influence HRV, or previously admitted to the hospital within 30 days were excluded. Cardiac autonomic functions were evaluated by frequency domain indices of HRV. Multiple logistic regression was used to assess the association between UR and HRV indices adjusted for age and length of hospitalization.

**Results:**

A total of 78 patients met the inclusion criteria and 15 of them were readmitted within 14 days after discharge. The risk of UR was significantly higher in patients with lower levels of total power (OR = 1.39; 95% CI = 1.04-2.00), low frequency power (LF) (OR = 1.22; 95% CI = 1.03-1.49), high frequency power (HF) (OR = 1.27; 95% CI = 1.02-1.64), and lower ratios of low frequency power to high frequency power (LF/HF ratio) (OR = 1.96; 95% CI = 1.07-3.84).

**Conclusion:**

This is the first study to evaluate the association between frequency domain heart rate variability and the risk of UR in geriatric patients. Frequency domain heart rate variability indices measured on admission were significantly associated with increased risk of UR in geriatric patients. Additional studies are required to confirm the value and feasibility of using HRV indices on admission as a non-invasive tool to assist the prediction of UR in geriatric patients.

## Background

With the ageing population and the shortage of acute hospital beds, the cost of unplanned readmission (UR) among elderly patients to health service is high. Hospital readmission in 2004 accounted for about 17% of the total Medicare's hospital bill in the United States [1]. UR is also associated with high levels of psychological distress and frustration in patients and their relatives. In addition, repeated hospital admissions have been suggested to lead to dependency and result in a self-perpetuating cycle [2].

Readmission to hospital has often been considered as an indicator of quality of hospital care. It can be defined as admission to the same hospital within a specified period time since discharge from a hospital. Various time period such as 7, 14, 15, 28, 30 days after discharge have been reported in the literature [3-6]. Previous studies have suggested that increased UR was associated with frailty, severe disability [3], age, lack of a family physician, reduced social support, heart disease, diabetes [7], tumor, dementia [8], anemia [9,10], falls, urinary incontinence, less supportive living arrangement [11], length of prior admission [8,12], numbers of medical problems, numbers of previous admission [13], activities of daily living [14,15], Bathel Index, Mini-Mental State Examination, and the triceps skin fold thickness [8]. Although some the above factors are caused by patients' frailty and progression of their chronic diseases, it has been estimated that up to 48% of all readmissions have been judged to be preventable by measures such as better predischarge assessment, patient education, and postdischarge care [16]. Therefore, by identifying factors associated with UR, particularly those that can be obtained non-invasively on admission, geriatric medical units can allocate available resources to allow a more effective management of any high-risk UR patients.

Frequency domain heart rate variability (HRV) analysis has been used as a non-invasive tool to assess the status of cardiac autonomic functions. The standards of measurement, physiologic interpretations, and clinical applications of HRV analyses have well been documented since 1996 [17]. Recent studies indicated that HRV reflects the dynamic operations of neural regulation of the heart. It can serve as signal markers for physiological or pathological events in healthy vegetarian women [18], in patients with brain death [19] or with terminal hepatic cancers [20], and those in intensive care unit [21]. Therefore, we conducted a prospective study to explore whether cardiac autonomic functions could be used as a predictor for UR in geriatric patients. The objective of this study is to investigate the association between frequency domain indices of heart rate variability on admission and 14-day UR in geriatric patients.

## Methods

The study was approved by the Human Research Ethics Committee of the Buddhist Dalin Tzu Chi General Hospital and all subjects gave informed consent. Patients admitted to the geriatric ward at the Buddhist Dalin Tzu Chi General Hospital, Chiayi, Taiwan during the period from July 2006 to June 2007 were eligible for inclusion the study.

In this study, UR was defined as readmission to the hospital within 14 days after being discharged from an earlier hospital stay. A readmission period of 14 days was chosen in this study over other durations because it is the standard duration used by the Taiwan's Bureau of National Health Insurance for monitoring the quality of hospitalization cases [22].

Patients with invasive tubes, such as tracheostomy tube, Foley's tube, and naso-gastric tube; heavy smokers; heavy alcohol drinkers; or taking medications that might influence HRV, such as alpha-blockers or beta-blockers antihypertensives were excluded from the study. Patients who were discharged from the study hospital between 15 and 30 days were excluded from the study to act as a wash-out period in order to minimize the potential influences from their previous hospital stay. In addition, patients who had multiple 14-day readmissions during the study period were excluded. Therefore, each patient can only be considered as either an index admission or a readmission, but not both, in the data analysis.

Demographic characteristics, biochemical test results, and clinical symptoms and signs of the patients were collected by a team comprised of physicians and nurses within 24 hours of admission. Biochemical test variables included white blood cell (WBC) counts, blood urea nitrogen (BUN), blood glucose, and serum glutamic oxaloacetic transaminase (GOT). Clinical symptoms and signs included blood pressure, heart rhythm, Glasgow Coma Scale scores, and activity levels of the patients.

Five-minute electrocardiography (ECG) recordings were performed on admission between 14:00 and 16:00 hours with patients in supine position. ECG signals were taken by precordial leads and were recorded using a 12 bit analog-digital converter (PCL-818, Advantech, Taiwan) with a sampling rate of 1024 Hz. Each QRS complex was identified. The R point of each valid QRS complex was defined as the time point of each heart beat, and the interval between two R points (R-R interval) was estimated as the interval between the current and latter R points (PPI). Frequency domain analysis of PPI was performed using the nonparametric method of fast Fourier transform (FFT). For each time segment (288 seconds, 2048 data points), the algorithm estimated the power spectral density on the basis of FFT. The resulting spectrum was corrected for attenuation resulting from the sampling and Hamming window. The power spectrum was subsequently quantified by integration into frequency domain indices including total spectrum power (TP), high frequency power (HF, 0.15-0.4 Hz), low frequency power (LF, 0.04-0.15 Hz), and the ratio of lower frequency power to high frequency power (LF/HF ratio). The power content of the HF component corresponds to respiratory sinus arrhythmia and is modulated solely by the parasympathetic nervous system. The power content in the LF component is modulated jointly by the sympathetic and parasympathetic nervous systems. The LF/HF ratio is used to reflect sympathovagal balance. The power of the very low frequency component (0-0.04 Hz) was not calculated because it was reported to be unreliable over short recording periods [17].

Data entry and analysis were performed using commercially available software (SAS, version 9.1.3, SAS Institute Inc., Cary, NC, USA). All statistical assessments were two-sided and statistical significance was set at the 0.05 level. The basic characteristics between UR and non-UR patients were analyzed using nonparametric Mann-Whitney test for continuous variables or Fisher's exact test for categorical variables. The frequency domain HRV indices were natural-logarithm transformed to reduce skewness of the data distribution [18]. Multiple logistic regression analyses were conducted to analyze the association between the frequency domain HRV indices and UR adjusted for age and length of hospitalization. Hosmer and Lemeshow Goodness-of-Fit Test was conducted to determine the adequacy of the fitted logistic models.

## Results

A total of 131 patients were admitted to the geriatric ward of the study hospital during the period from July 3, 2006 to June 30, 2007. Fifty-three patients were excluded from the study and they included 30 patients who were discharged from the hospital within last 15 to 30 days, 6 patients who died during admission course, 14 patients who were lost to follow up, and 3 patients who were on alpha or beta blocker's antihypertensives. Therefore, 78 patients were included in the analysis. Within the study period, 19.2% of the patients (15 of 78) were readmitted within 14 days after discharge from their index admission.

The basic characteristics of the patients are shown in Table 1. There were no significant differences in sex, age, lengths of hospitalization, proportions with hypertension and diabetes, distributions of diagnosis on admission, activity levels, Glasgow Coma Scale scores, blood urea nitrogen levels, glucose levels, and serum GOT levels between UR and non-UR patients. Counts in WBC before discharge was significantly higher (p = 0.027) in UR patients than those in non-UR patients. The two most frequently diagnoses at index admission were pneumonia and urinary tract infection. Regarding their activity levels in the hospital, most patients (82.1%) were bedridden.

**Table 1:** The basic characteristics of study subjects

Variable		Unplanned Readmission *n *= 15 n (%)	Non-unplanned readmission *n *= 63 n (%)	*p-*value
Sex	male	5 (33.3)	28 (44.4)	0.434
	female	10 (66.7)	35 (55.6)	
Age (years)		80 (72, 90)*	81 (73, 86)	0.929
Length of hospitalization (days)		10 (6, 16)	9 (7, 13)	0.541
Hypertension		9 (60)	41 (65)	0.713
Diabetes		7 (47)	19 (30)	0.223
Diagnosis at index admission				
	pneumonia	9 (60)	25 (40)	0.154
	urinary tract infection	8 (53)	44 (70)	0.223
	electrolyte imbalance	3 (20)	9 (14)	0.582
	gastro-intestinal tract bleeding	1 (7)	2 (3)	0.527
	cerebral vascular accident	2 (13)	8 (13)	0.947
Activity level				0.288
	can walk in hospital room	1 (7)	3 (5)	
	on bed and can change position	0	9 (14)	
	bedridden	14 (93)	50 (79)	
Glasgow Coma Scale		12 (7, 14)	10 (8, 14)	0.880
WBC before discharge (×10^3^/μl)		8.8 (6.7, 10.0)	6.8 (5.6, 8.5)	0.027
Blood urea nitrogen (mg/dL)		25 (13, 32)	16.5 (13.0, 29.0)	0.584
Blood glucose (mg/dL)		142 (110, 239)	128 (105, 164)	0.302
Serum GOT (mg/dL)		20 (14, 23)	22 (16, 32)	0.212

WBC = white blood cell; GOT = glutamic oxaloacetic transaminase

* continuous variables were expressed as median (first quartile, third quartile)

Comparison of frequency domain HRV indices between UR and non-UR patients is showed in Table 2. Levels of total power, LF, HF, and LF/HF were significantly higher in non-UR patients compared to UR patients. Table 3 showed the odds ratios of the associations between HRV indices and non-UR. Odd ratios greater than 1 mean that lower level of HRV indices were associated with increased risk of UR. The risk of UR was significantly higher in patients with lower levels of total power (OR = 1.39; 95% CI = 1.04-2.00), low frequency power (LF) (OR = 1.22; 95% CI = 1.03-1.49), high frequency power (HF) (OR = 1.27; 95% CI = 1.02-1.64), and lower ratios of low frequency power to high frequency power (LF/HF ratio) (OR = 1.96; 95% CI = 1.07-3.84).

**Table 2:** Comparison of frequency domain heart rate variability indices between unplanned readmission and non-unplanned readmission in geriatric patients

Variable	Unplanned Readmission *n *= 15	Non-unplanned readmission *n *= 63	*p*-value
Total power, ln(ms^2^)	5.57 (4.07, 6.34)	6.80 (4.68, 8.58)	0.021
LF power, ln(ms^2^)	3.85 (0.45, 5.07)	5.54 (2.48, 7.04)	0.013
HF power, ln(ms^2^)	3.51 (1.17, 4.29)	4.72 (3.17, 6.03)	0.035
LF/HF ratio	-0.43 (-0.71, 1.19)	0.76 (-0.23, 1.48)	0.025

LF = low frequency; HF = high frequency

* variables were expressed as median (first quartile, third quartile)

**Table 3:** Results of multiple logistic regression of frequency domain heart rate variability indices associated with non-unplanned readmission in geriatric patients

HRV indices	Odds ratio	95% confident interval	*p*-value
Total power, ln(ms^2^)	1.39	1.04-2.00	0.046
LF power, ln(ms^2^)	1.22	1.03-1.49	0.032
HF power, ln(ms^2^)	1.27	1.02-1.64	0.046
LF/HF ratio	1.96	1.07-3.84	0.036

LF = low frequency; HF = high frequency

*All models were adjusted with age and length of hospitalization.

*P-values of Hosmer and Lemeshow Goodness-of-Fit Test: Total power = 0.622, LF = 0.078., HF = 0.727, and LF/HF ratio = 0.723.

## Discussion

The present prospective study is the first study in evaluating the association between cardiac autonomic functions as measured by frequency domain HRV and the risk of UR in geriatric patients. Lower levels of TP, LF, HF, and LF/HF ratio on admission were significantly associated with increased risk of UR in geriatric patients. Using HRV indices for UR prediction has the advantages of accessibility, ease of standardization, and noninvasiveness. There were no previous studies available in the literature that explored the use of HRV indices in UR prediction in geriatric patients. At present, UR prediction depends mostly on screening of clinical factors, which subjects to variations associated with the experience and judgment of clinicians. On the other hand, the use of objective HRV indices can minimize the influences of individual variations.

Frequency domain HRV analysis is a noninvasive tool for measuring cardiac neural regulation. It has been well established that the HF component of HRV is equivalent to the vagal regulation of the heart whereas the LF/HF ratio can be considered to reflect sympathetic modulations. The levels of LF and total power can reflect the strength of autonomic regulation on the heart [17]. Since the autonomic neural control may play an important role in regulating cerebral circulation [23], greater strength of cardiac autonomic functions imply a better cerebral circulation. This may explain the association between greater strength of cardiac autonomic functions, as reflected by the increased levels of TP and LF, and a lower risk of UR in the present study. Previous studies have shown that HRV could reflect the body's adaptability to a stressed physiologic state [24]. Therefore, it is plausible that HRV can also reflect the general body reserve of geriatric patients and in turn, their risk of UR. The exact mechanisms linking HRV and UR in geriatric patients warrant additional exploration.

Regarding the laboratory data, the levels of WBC before discharge from the hospital was significantly higher in UR patients compared with those in non-UR patients. The increased in WBC counts might reflect a subclinical inflammatory status in the UR patients. Other biochemical data were not significantly different between the two groups of patients. This is in contrast to a previous study designed to examine the rate of UR within the most recent postoperative year for heart transplant patients. The authors reported that BUN and creatinine levels were significantly higher in readmitted patients than in patients who were not [25].

A few limitations in this study should be noted. First, we excluded patients who had multiple readmissions from the study and therefore, our results are applicable only to the case of single unplanned readmissions. Whether cardiac autonomic functions are associated with UR in patients with multiple readmissions will require further investigations. Second, our findings from a single regional hospital may not be representative for other geriatric wards with different sources of patients. Most of our patients came from nursing homes rather than from their own homes. Third, the loss of 14 patients to follow up raised the concern that their readmission might have occurred in other hospitals. However, these patients were residents of nursing homes which had contracts with the study hospital to provide health services to their residents. Therefore, readmission to other hospitals was unlikely to have happened.

## Conclusions

This is the first study to evaluate the association between cardiac autonomic functions as measured by frequency domain HRV and the risk of UR in geriatric patients. Lower TP, LF, HF, and LF/HF ratios on admission were significantly associated with increased risk of UR in geriatric patients. Additional studies are required to confirm the value and feasibility of using HRV indices on admission as a non-invasive tool to assist the prediction of UR in geriatric patients.

## Competing interests

The authors declare that they have no competing interests.

## Authors' contributions

JKC conceived the research questions, designed the study and drafted the initial manuscript. CHF was involved in data analysis and revisions. TBJK was involved in preparatory field works and data collection. MK was involved in the interpretation of data and revisions of the manuscript for publication. All authors revised, read and approved the final manuscript.

## Pre-publication history

The pre-publication history for this paper can be accessed here:

http://www.biomedcentral.com/1471-2458/11/137/prepub
